# Structure of the Ebola virus glycoprotein spike within the virion envelope at 11 Å resolution

**DOI:** 10.1038/srep46374

**Published:** 2017-04-11

**Authors:** Daniel R. Beniac, Timothy F. Booth

**Affiliations:** 1 Viral Diseases Division, National Microbiology Laboratory, Public Health Agency of Canada, Winnipeg, Manitoba, R3E 3P6, Canada; 2 Department of Medical Microbiology and Infectious Diseases, Max Rady College of Medicine, University of Manitoba, Winnipeg, Manitoba, R3E 0W3, Canada

## Abstract

We present the structure of the surface Ebola virus (EBOV) trimeric glycoprotein (GP) spike at 11 Å resolution, *in situ* within the viral plasma membrane of purified virus particles. GP functions in cellular attachment, endosomal entry, and membrane fusion to initiate infection, and is a key therapeutic target. Nevertheless, only about half of the GP molecule has yet been solved to atomic resolution, excluding the mucin-like and transmembrane domains, and some of the glycans. Fitting of the atomic resolution X-ray data from expressed, truncated deletion constructs within our 11 Å structure of the entire molecule demonstrates the relationship between the GP1-GP2 domains, the mucin-like and transmembrane domains, and the bilaminar lipid envelope. We show that the mucin-like domain covers the glycan cap and partially occludes the receptor binding sites prior to proteolytic cleavage. Our structure is also consistent with key antibody neutralisation sites on GP being accessible prior to proteolysis. Based on the findings of us and others, GP-mediated binding may create an angle of 18 degrees between the planes of viral and endosomal membranes.

Ebola viruses (EBOVs) cause periodic outbreaks of severe hemorrhagic disease^1^. The recent deadly outbreak in West Africa (2013–2016) was the largest in history, with 28,000 cases and 11,000 deaths^2^. EBOV has a striking, filamentous structure: the helical nucleocapsid acquires an envelope by budding from the plasma membrane, a process driven by the VP40 matrix protein. The viral envelope contains spikes consisting of the glycoprotein (GP) trimer^3^,^4^,^5^. This GP molecule achieves the combined functions of attachment to host cells, endosomal entry, and membrane fusion^6^,^7^,^8^,^9^,^10^,^11^,^12^. In the current study, we present the structure of the surface glycoprotein (GP) spike at 11 Å resolution, *in situ* within the viral plasma membrane, determined by cryo-electron microscopy (cryo-EM). As a class I fusion protein, EBOV GP plays key roles in cellular attachment, and entry of the virus into host cells, and is a key target for immune and therapeutic approaches^13^,^14^,^15^,^16^,^17^,^18^. There is extensive glycosylation, particularly in the mucin-like domain. The GP is cleaved into two disulfide-linked proteins, GP1 and GP2: GP1 contains the receptor binding and mucin-like domains. The endosomal receptor for GP is the Nieman-Pick disease type C1 (NPC-1)- protein^10^,^19^. Cathepsin cleavage of GP in the endsome is necessary for exposure of the NPC-1 binding site and subsequent fusion: binding to the receptor is thought to trigger fusion^13^,^14^,^17^,^20^,^21^,^22^. GP2 is bound to the viral envelope by the transmembrane domain and contains the fusion peptide which achieves membrane fusion^19^,^20^,^21^,^22^,^23^,^24^,^25^. Recently an unliganded X-ray crystallography structure of the EBOV GP was determined^14^. The latter is the largest and most detailed structure of the EBOV GP described so far, however certain domains are still missing, due to the need to truncate the transmembrane and certain glycosylation sites in order to achieve crystallization. We have docked these new atomic resolution structures within our 11 Å cryo-EM structure of the entire EBOV GP trimer imaged in the viral membrane, to generate a variable resolution structural map of the entire integral-membrane spike.

EBOV has a single-stranded negative-sense 19 kb RNA genome encoding eight structural proteins, including the nucleoprotein (NP), virion proteins (VP24, VP30, VP35, and VP40), polymerase protein (L), the transmembrane glycoprotein (GP) and a soluble glycoprotein (sGP)^1^. The GP spike is the only surface protein in EBOVs, unlike some other enveloped viruses, for example paramyxoviruses, which have separate proteins that perform the viral attachment and fusion functions. In EBOV the GP is solely responsible for both of these functions and the presence of a single species of spike molecule simplifies analysis by cryo-electron microscopy and image processing. Previous structural investigations of the entire GP trimer have relied upon recombinant expression of truncated mutants without a transmembrane domain, or as smaller sub-domains of the GP molecule, or as artificial virus-like particles (VLPs)^15^,^16^,^18^,^24^,^26^,^27^,^28^,^29^. The structure presented in the current investigation is based solely on data from the entire glycosylated GP on the surface of EBOV, using virions purified from EBOV infection in cell culture, and not recombinant expressed versions of the GP spike.

## Results and Discussion

**Figure 1:**
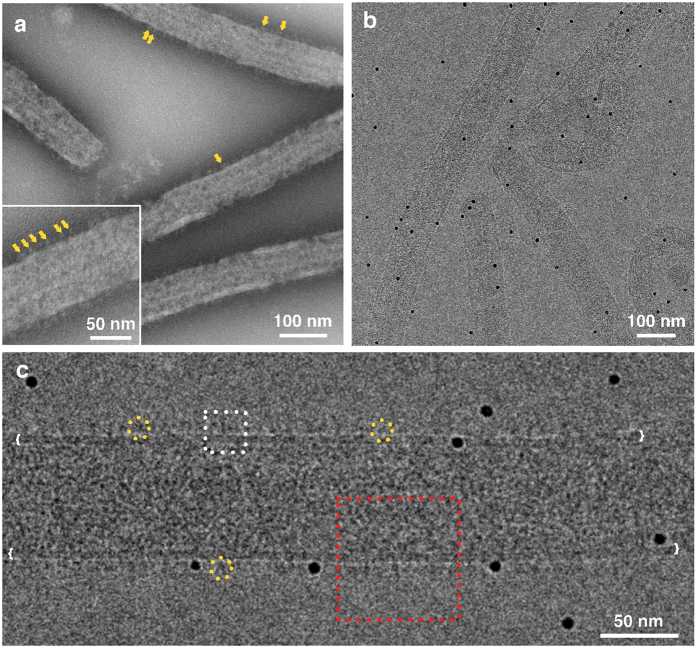
Electron microscopy of (**a**) Negative stained EBOV, (inset showing the virus at higher magnification). A defocus of 2–3 microns was used, thus spikes are visible in low contrast. The yellow arrows point to individual GP spikes. (**b**) Cryo-EM of EBOV, black “dots” are 10 nm colloidal gold added for focusing. (**c**) Higher magnification showing individual GP spikes (yellow circles), the viral envelope is indicated by white brackets. The red square shows the relative size of the half-diameter images used for the image processing symmetrical analysis shown in Supplementary Fig. S3. The white square shows the relative size of the images that were used for the image processing data in shown Fig. 2 and Supplementary Fig. S2.

To establish a more definitive structure for the native spike within the EBOV particle, we analysed purified *bona-fide* EBOV in order to image the GP spike within the virion envelope (Fig. 1a,c). These GP spike images were analysed using the single particle method only (Fig. S1,S2), as a comparison to structures previously obtained by us and others using tomographic methods. Discrepancies had been observed between the structures of the entire, untruncated EBOV GP determined using material produced with differing heterologous expression systems, and between structures obtained using alternative tomographic or single-particle three-dimensional image processing methods 4,28. Due to safety concerns, the virus preparation was treated using paraformaldehyde crosslinking (after centrifugation) in a protocol that has previously been shown to preserve protein and lipid structures^4^,^30^. Ebola virions are flexible, and viral filaments are frequently curved when prepared in the frozen-hydrated state for cryo-electron microscopy (Fig. 1b). Therefore, regions of virions that were as straight as possible were selected for image processing (Fig. 1c). Our data included 32,960 individual spike images for single particle analysis. In addition, 29,976 images were selected for reference-free analysis of the half-diameter of EBOV to investigate the spatial distribution of the GP spikes, as well as the periodicity and symmetrical relationships between GP and the matrix protein VP40 in the envelope, and the underlying nucleocapsid layer (Figs 1c and S3).

**Figure 2:**
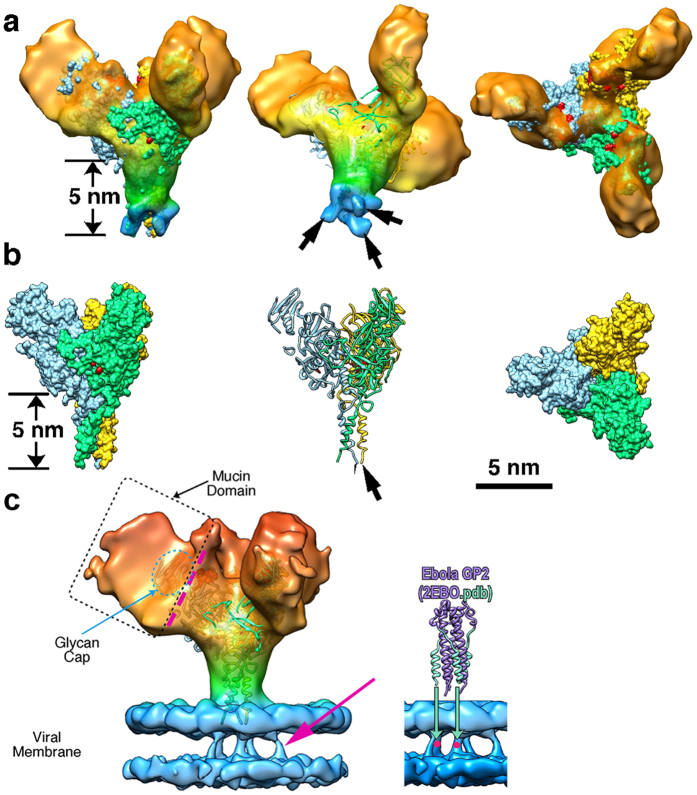
3D structure of the EBOV spike. (**a**) GP trimer with the docked GP1-GP2 structure 5JQ7 shown from various perspectives. The colour scheme for the 3D reconstruction is as follows: orange, distal end of GP spike; yellow, body of GP spike; green, stalk of GP spike; and blue, integrated in viral envelope. The three GP1-GP2 subunits in 5JQ7 are coloured blue, green, and yellow, and toremifene in red. (**b**) Surface and ribbon displays of 5JQ7 for comparison with (**a**): C-terminal ends of GP2 are indicated by arrows. (**c**) Transmembrane densities visible in the low-pass 13.4 Å filtered reconstruction (red arrow, on left) line up with the heptad repeat alpha-helices of the docked GP2 core structure 2EBO (right: red dots).

**Figure 3:**
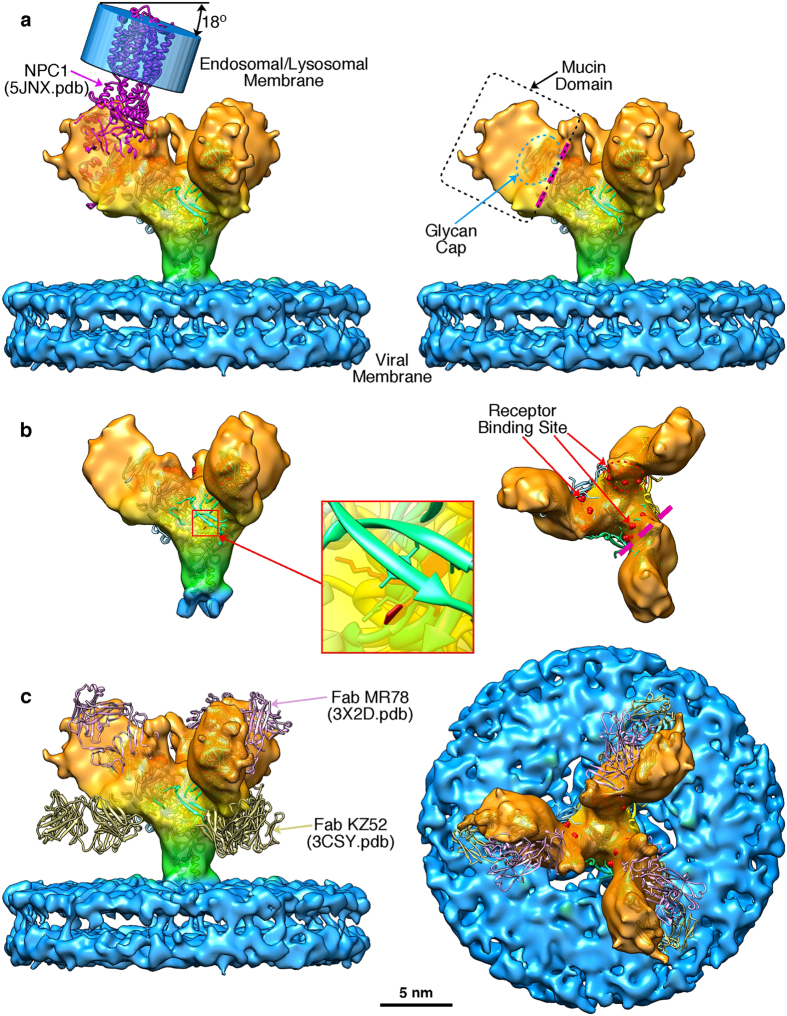
Merging cryo-EM and atomic resolution 3D structures. (**a**) The atomic resolution structures 5JQ7 and 5JNX (NPC1-GP, magenta) were docked within the Ebola GP using Chimera (GP trimer subunits coloured in green, blue and yellow; NPC1-GP in magenta). The cryo-EM reconstruction is presented at a threshold to show the viral envelope, and using the same colour code as in Fig. 2. (**b**) The GP spike reconstruction is presented at a threshold equivalent to its molecular mass to illustrate the tight fit of 5JQ7. The inset shows the location of toremifene (red), and the residues of the receptor-binding site are coloured red. (**c**) The atomic resolution structures of the neutralizing antibodies KZ52 Fab (3CSY, beige), MR78 Fab, (3X2D, pink) were docked within the Ebola GP using Chimera.

**Figure 4:**
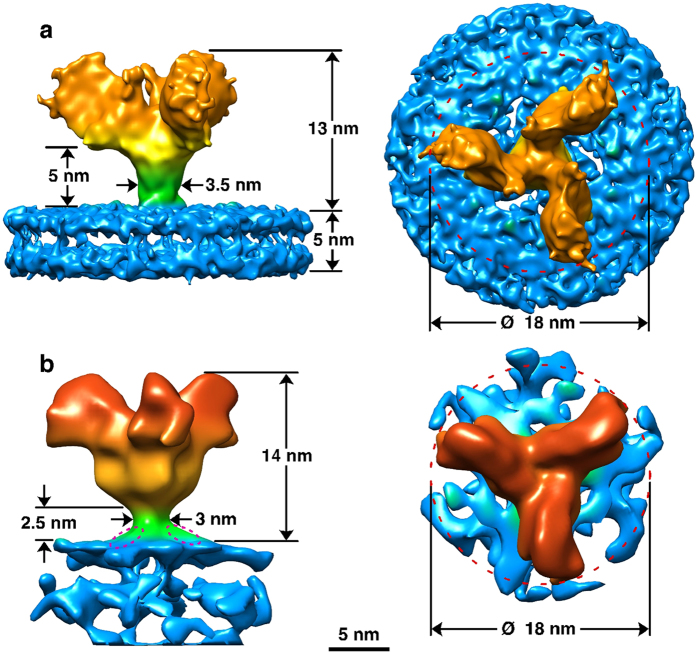
Comparison of Ebola spike structures bound to the viral membrane. The single particle derived structure presented in this investigation (**a**) is compared to a structure calculated using sub-tomogram analysis (**b**). The cryo-EM reconstructions are presented using the same colour code as in Fig. 2.

A 3-D structure for the GP spike trimer *in situ,* within the viral envelope, with a resolution of 11 Å was calculated (Figs 2 and 3, Supplementary Fig. S4) using the projection-matching procedure on masked images^31^,^32^. We were able to dock the atomic structure of the EBOV GP (5JQ7) containing the full-length GP1 and GP2 domains, but with the mucin-like domains truncated (Fig. 2b). This clearly demonstrates a number of features of the viral-derived spike, including the structure and arrangement of the mucin-like domains with respect to the GP1-GP2 structure. In particular, the base of the spike, consisting of the GP2 fusion domains with the heptad repeat motifs, fits extremely well: the atomic resolution structure 5JQ7 fills the volume of our 3-D cryo-EM structure (Fig. 2a). The closeness of fit, especially in the stalk or “neck” area of the GP trimer, is apparent when the spike structure is displayed at a density where the 5 nm thick bilayer nature of the virion envelope is clearly visible (Figs 2a,c and 3a) and also when the surface is truncated to a level where the volume approximates to a molecular weight of 310 kDa (Figs 2 and 3b). The alpha-helices of GP2 are visible at the base of the spike, (Fig. 2a) and when the reconstruction is displayed at a slightly higher contour level, densities that cross the interior of the membrane are visible (Fig. 2c). Since the N-terminal ends of the docked alpha-5 GP2 domains (2EBO^24^) appear to line up with these transmembrane densities, the latter may be an indication of the putative hydrophobic alpha-helical transmembrane regions of GP2 (Fig. 2c). Using the predicted mass of the GP (as measured by gel electrophoresis of virion-derived GP^33^) we adjusted the volume of the 3-D structure in Fig. 2, using a value of 0.8 Da/Å^3^ as an approximate density for protein^34^. Since the structure 5JQ7 is 163 kDa^35^, the truncated mucin-like and transmembrane domains, including glycans, represent ~150–170 kDa, approximately half the mass of the spike. When viewed from above, the GP spike looks like a three-bladed propeller (Figs 2a and 3b). The length of the three “blades” encompasses a circle that is 18 nm in diameter. When viewed from the side, the GP spike has a stalk region adjacent to the viral envelope, which then spreads out to the upper part of the GP spike. The toremifene-binding “pocket” or “tunnel” identified in 5JQ7 is near the surface of the 3-D EM structure, on the side of the stalk at the base of the propellers (Figs 2a and 3b). At the base of the stalk in Fig. 2a, the three heptad repeat helices at the base of GP2 fit neatly into three strands visible in our EM 3-D structure, that appear to penetrate the virion membrane (Figs 2a and 3a). Also, when viewed from the top, each blade has a smaller nub closer to the 3-fold axis that protrudes distally (Figs 2a and 3b). Using the 332 kDa contour level, these nubs correspond closely with the receptor binding site, covering most of the residues known to be involved in binding, as well as protruding adjacent to the glycan cap regions. Each propeller blade, which is known to contain the mucin-like domain^28^, completely covers the site known to bind to the NPC-1 receptor (Fig. 3b). Thus approximately half of the mass in our structure, external to the envelope, are the mucin-like domains. When the density map is contoured to a level that removes the lipid bilayer from display, the stalk still conforms closely to the surface of the atomic resolution structure, whereas there distal ends of the blades and the glycan cap “nubs” are slightly truncated. This indicates that these regions of the structure probably have a lower density, consistent with their being highly glycosylated, as predicted by the amino acid sequence of the mucin-like domain.We also docked the 5JNX structure^36^, which includes part of the cleaved glycoprotein (GPcl) in complex with full-length human NPC1, and overlaid the atomic resolution structure onto one monomer using the program module “Fit in Map” in UCSF Chimera (Fig. 3a). This indicates that the mucin-like domain, (all of the unoccupied density remaining in the 3-D EM structure when GP1-GP2 are fitted) completely covers the glycan cap, with the nub at the side of the propeller covering most, if not all, of the receptor binding site (Fig. 3a), indicating that removal or cleavage of the mucin-like domain (probably including the nub), as well as the glycan cap, may be prerequisites for receptor binding to be achieved. The docked 5JNX structure also includes the transmembrane region of NPC1, and thus we aligned the approximate plane of the plasma membrane when the GP spike docks with the receptor NPC1, shown in transparent blue in Fig. 3a. In the absence of any bending of endosomal membrane to accommodate the contour of the GP trimer and NPC1 complex, the plane of the plasma membrane would make an angle of about 18 degrees from the viral membrane when the spike is perpendicular to the viral envelope (Fig. 3a). This is consistent with the suggestion by Gong *et al*.^36^ that perhaps only one NPC1-receptor binding site out of three on each trimer can be occupied at once, and that binding to more than one receptor might be precluded due to stearic interference. The possible line of cleavage delineating the mucin-like domain and glycan cap from the rest of the trimer is shown in Fig. 3a and b. Our structure suggests that the mucin-like domain and the glycan cap might be cleaved at the same time, since the latter is entirely within the density of the putative mucin-like domain, and is consistent with a molecular weight for the mucin-like domain of about 50 kDa, which is similar to that predicted by the sequence as well as gel electrophoresis, although the length of the sugar side-chains is still unknown. In addition, we fitted the atomic-resolution Fab antibody-GP structures that bind to each of the two major EBOV GP neutralising epitopes that have been previously investigated, MR78 (3X2D^15^) and KZ52 (3CSY^18^): (Fig. 3c). This indicates that, within the limitations of the resolution achieved in our 3-D structure of the spike *in situ* in the envelope, the putative mucin-like domain (likely to consist of the propeller blade and possibly the nub features described here) is unlikely to interfere or stearically hinder either of these antibody neutralisation sites, consistent with neutralisation being possible prior to cleavage of the mucin-like domain in the endosome. It is clear that our structure differs from the 3-D EM structures of the heterologously-expressed Ebola GP spike observed *in situ* in the plasma membrane previously published by us and others (Fig. 4). Both of these structures included engineered expressed material using mutated or partially sequence-truncated proteins made in virus-like particles. The spike structure of Beniac *et al* imaged within the virus-like particles was somehow clipped, since this should have included the mucin-like domains: in addition, tomography was combined with single-particle analysis, which may have distorted the results^4^. The structure that we report here is based entirely on the well-accepted method of single-particle analysis using projection matching, and is broadly similar to that published of expressed GP in virus-like particles using tomography^28^,^29^: there are noticeable differences in shape and size of the spike, as well as in resolution (Fig. 4). The previously determined atomic resolution structures of GP appear to fit well within the cryo-EM structure we report here (Figs 2 and 3) and the resolution, although modest (11 Å), is an improvement compared to ~25 Å for the structures generated from tomography alone that appear to have a slightly different shape (Fig. 4). While our structure shows a spike length of 13 nm and a stalk length of 5 nm and width of 3.5 nm, the tomographic structures show a spike length of 14 nm, with a more pinched, shorter and more narrow stalk region of 2.5 nm in length and 3 nm width, while the virion envelope at the base also appears to be sloped away from the GP stalk (Fig. 4). Our structure clearly delineates the 5 nm bilayer of the virion envelope (that has an internal spacing of about 35 Å, Fig. 4a) as well as the alpha helices of the heptad repeat domains (that have a diameter of about 12 Å) and the nub feature at the base of the propellers, whereas these features were not discernable in the previous structures^28^,^29^ (Fig. 4b). These inconsistencies could be due to the differences between the expressed and viral materials, the latter of which was used in the present report. Differences in the methods used could also be significant, and a factor in the improved resolution was the use of an optimally sized T-shaped mask, and the selection of straight regions of the virion membrane for analysis (Supplementary Figure S3). This allowed accurate alignment and selection of images taken at right-angles to the three-fold axis of the spikes, while getting an excellent coverage of side views, since the spikes are randomly distributed on their 3-fold axes, using Euler angles of 0-120 degrees of rotation around the Z-axis (since it is a trimer). Thus, we avoided the “missing wedge” of information associated with tomography, where the constraint of tilt angle limits the angular distribution of views. Our analyses did not reveal any longitudinal symmetry or well-ordered periodicity of the GP spikes along the axis of the virion filament, showing that the spike arrangement of virions may not follow a rigid symmetry. Nor was any longitudinal symmetry apparent in the virion VP40 matrix layer (although a 5 nm lattice spacing in the VP40 layer has been observed in images showing a perpendicular view of the VLP membrane^4^). We have shown that the GP spikes of EBOV virions likely have an inconsistent or variable spacing, similar to that previously shown for EBOV VLPs (Supplementary Fig S3). We also confirmed previous results^4^ showing that the nucleocapsid layer of virions maintains a consistent ordered longitudinal spacing of about 7 nm (Supplementary Fig S3), despite the curves and bends of the filament. Thus any contacts between GP and VP40 and/or the nucleocapsid proteins may be variable and non-equivalent: or if a preferred alignment and stoichiometry exists, it was not detectable by cryo-EM of whole virions with our current data.

In conclusion, the current structure is consistent with the mucin-like domain occluding the glycan cap and receptor-binding region, such that cleavage is required for the functioning of the latter in the endosome to reveal the receptor binding site on GP1^13^,^17^,^20^,^21^,^22^. It is likely that the cleavage of the glycan cap also includes removal of both the “propeller” and “nub” structures described here. It is clear that the density in our structure partially covers the receptor binding domain, and would likely inhibit NPC-1 binding until cleavage of the mucin-like domain occurs. Further high resolution studies of the viral-derived GP structure and virion particles are needed to answer these questions, and for progressing knowledge of EBOV morphogenesis. In future studies, analyses of the spatial arrangement of the spikes in the membrane, the structural arrangement of the transmembrane and cytoplasmic domains, and analysis of flexible, quasi-equivalent connections between the envelope matrix protein VP40 and the nucleocapsid, will all be important to further our understanding of how these viral components function in the replication cycle of EBOV.

## Methods

### Cells and viruses

Zaire Ebola virus (Mayinga strain) was produced in Vero (E6) culture and purified and concentrated by centrifugation^37^. Viral preparations were characterised using polyacryalamide gel electropheresis and Western blotting. Samples were inactivated by using paraformaldehyde fixation (4%) followed by dialysis against PBS to remove the excess. Dialysis was carried out using a 0.5 ml Slide-A-Lyzer G2 cassette (10,000 MWCO: Thermo Scientific, USA). All virus culture and purification, and handling of infectious materials was carried out at the National Microbiology Laboratory within the biosafety level 4 laboratories in Winnipeg, Canada.

### Cryo-EM

Virus specimens (4 ul) were plunge-frozen on glow-discharged Quantifoil grids (2 um holes, at 1um intervals: Quantifoil, Germany). As a focussing aid, a BSA-colloidal gold suspension (10 nm particles: Aurion, The Netherlands) was added to virus preparations at a rate of 10 per cent by volume. Freezing was carried out using liquid ethane as a cryogen with a Vitrobot (Mark IV: FEI Company, USA). Images were taken at 200 kV with a Tecnai 20 G2 electron microscope (FEI) using a Gatan CT3500TR single tilt rotation specimen holder at −185 °C. Data was recorded with an Eagle 4K CCD camera (FEI Company, USA) at 50,000X magnification at 2–4 um defocus, with a dose of 10 electrons/Å^2^. This gave a pixel size of 2.15 Å at the CCD chip. Image recording used the automated low-dose TIA software (FEI Company, USA). Xplore3D data acquisition software (FEI Company, USA) was used to automate eucentric height and focus.

### Image processing

Particle selection was carried out using EMAN, and correction for contrast transfer function (CTF) was made using EMAN2^38^. Image processing and 3-D structures used the SPIDER and WEB programs^39^. Analyses were carried out on a Mac Pro 12-core computer (Apple Inc, with 2.93 GHzIntel Xeon Nehalem processors, 32GB RAM, running OS X 10.6.7) and on a Dell 6-Core Power Edge (R900, 64-bit Xeon X7460 2.67 GHz CPUs, 256GB RAM, running on Linux OS5.2). Correction of images for the CTF was carried out with the “e2ctf.py” module in EMAN2 This estimated defocus, and used phase-flipping to correct for the CTF. Spike images (n = 32,960), and half-width images of the virus (n = 28,976) were selected for image analysis. In all subsequent sections, SPIDER software was used for image processing except where stated otherwise. Methods are described and illustrated in Supplementary Figs S1–S3. Resolution of the final cryo-EM structure was estimated using the Fourier shell correlation 0.143 criterion^32^ (Supplementary Fig. S4).

### Single particle image analysis: half width of virus images

Specific image regions were analysed for single particle image processing. Two-dimensional masks were generated in the Canvas X software package (ACD Systems, Seattle, Washington, USA). Masks were imported as tiff files for image processing in SPIDER. Reference-free classification, to bring all images into register, was carried out with the EMAN software package. The class average was then used as reference to bring all the images into rotational and translational register. These pre-aligned images were then used with a series of masks for various regions of interest, both including the lipid bilayer, and with this region masked. EMAN was again used to perform a reference free classification, and the averages generated were processed in SPIDER to perform a multi-reference alignment. The results of these analyses were then applied to both masked and unmasked images to investigate effect of the masking procedure and periodicity of the different layers including the spikes, viral envelope and nucleocapsid.

### Single particle image analysis: GP Spike analysis

The masking described for 2-D image analysis was adapted to 3-D projection matching of EBOV spike images (Supplementary Figs S1–S3). For 3-D processing, images were pre-aligned to a global average by reference-free classification in EMAN, followed by data alignment to this average in SPIDER. Pre-aligned images were then masked with an upside down “T”-shaped mask, thus selecting image regions containing both the lipid bilayer and a single spike (Supplementary Fig S2). A dual set images was then sub-filed, one masked with the “T”-shaped mask, and the other unmasked. The 3D reconstruction of the GP spike was then generated by projection matching, using methods similar to those previously used with other viral spikes within their envelopes^4^,^40^. The main difference was the use of “T”- masked images for two-dimensional analysis and alignment, and unmasked images to apply the alignment parameters to generate new image averages. In addition a cylindrical mask was used on the 3-D reference volume. In both cases, the 2-D and 3-D masks selected data from the envelope and spike, and suppressed noise from adjacent spikes, as illustrated in Supplementary Figs S2 and S3.

### Structure visualisation and docking atomic resolution data

The 3D cryo-EM data, including docked atomic resolution structures 3CSY, 3X2D, and 2EBO were visualised with the Chimera software package^41^ (Computer Graphics Laboratory, University of California, San Francisco, supported by NIH P41 RR-010810).

**Data and materials availability.** The 3-D electron microscopy structure of the GP spike has been deposited into the Electron Microscopy Data Bank, www.emdatabank.org (accession number EMD-8630).

## Additional Information

**How to cite this article**: Beniac, D. R. and Booth, T. F. Structure of the Ebola virus glycoprotein spike within the virion envelope at 11 Å resolution. *Sci. Rep.* **7**, 46374; doi: 10.1038/srep46374 (2017).

**Publisher's note:** Springer Nature remains neutral with regard to jurisdictional claims in published maps and institutional affiliations.

## Supplementary Material

Supplementary Information
