# Supplementary material for: Structure of the Ebola virus glycoprotein spike within the virion envelope at 11 Å resolution
Source: Sci Rep. 2017 Apr 11;7:46374. doi: 10.1038/srep46374 (PMC5387728; doi:10.1038/srep46374)
Supplement: Supplementary Information [file srep46374-s1.pdf]

Supplemental Information

Structure of the native Ebola virus glycoprotein spike within the virion envelope at 11Å resolution.

Daniel R. Beniac[1], and Timothy F.Booth[1,2,*]

1 Viral Diseases Division, National Microbiology Laboratory, Public Health Agency of Canada,Winnipeg, Manitoba, Canada.

2 Department of Medical Microbiology and Infectious Diseases, Max Rady College of Medicine, University of Manitoba,Winnipeg, Manitoba, Canada.

*** E-Mail: tim.booth@canada.ca**

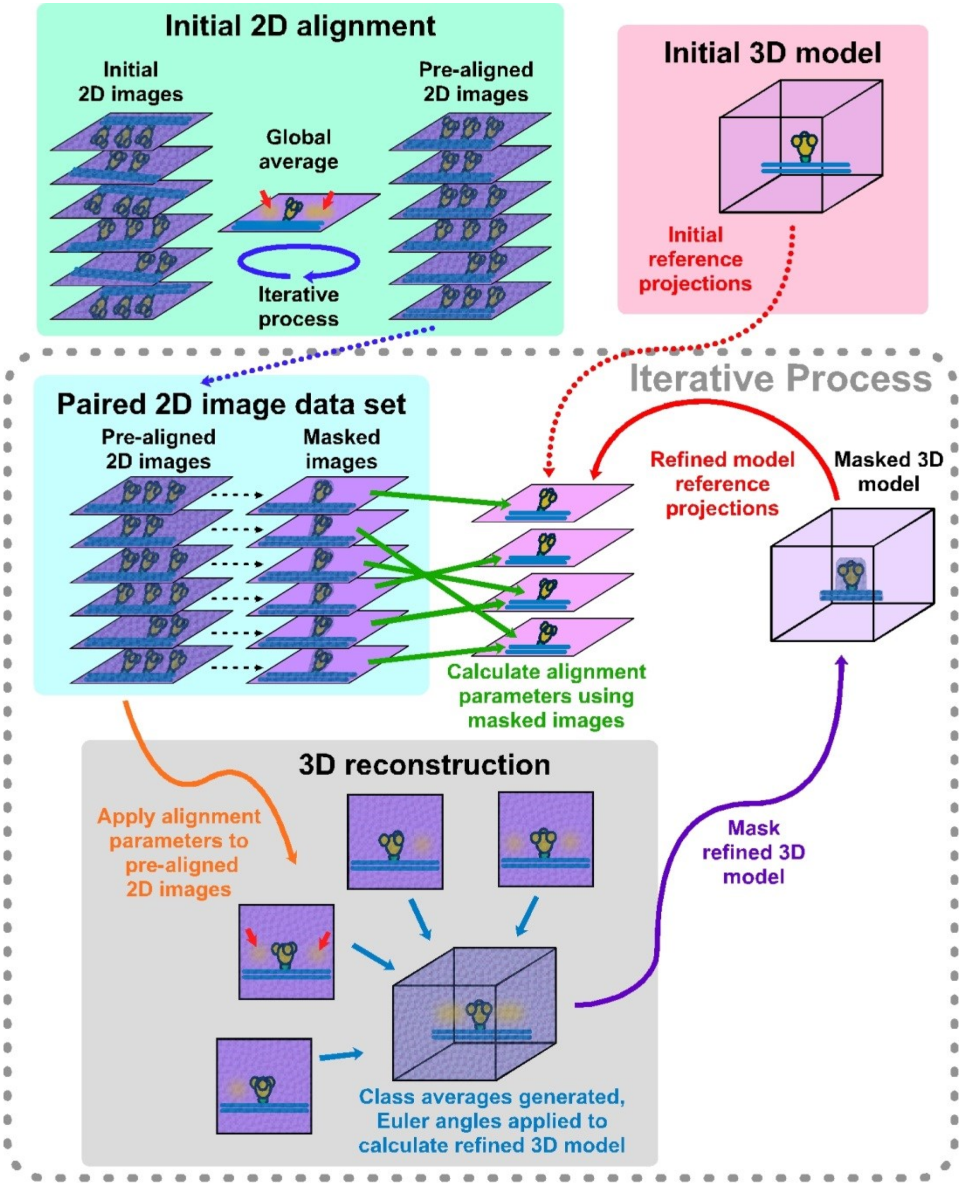

Supplemental Figure S1. An overview of the modified projection match procedure. All of the 2D images were first aligned to a global average of the data (green panel). With all of the images in the same orientation, a global mask was applied to each image in the data set. Thus, for each image, the selected area for analysis included a single GP spike, with a section of the viral lipid bilayer adjacent to it. A paired image set was generated, with masked and unmasked versions of each image (blue panel). The projection matching procedure is an iterative process. An initial 3D model (pink panel) was used to generate reference projections. These projections were then used to align the masked image data. The alignment parameters were then applied to the unmasked images, to generate class averages. Each class average is associated with a reference projection from the initial 3D model, and each of these projections have α,β, and γ Euler angles relating them to each other in three dimensional space. With these parameters applied, the images are back-projected to calculate a new 3D reference structure. Once the new structure is calculated, it is masked and then used as a source of reference projections, and the procedure is repeated through several iterations.

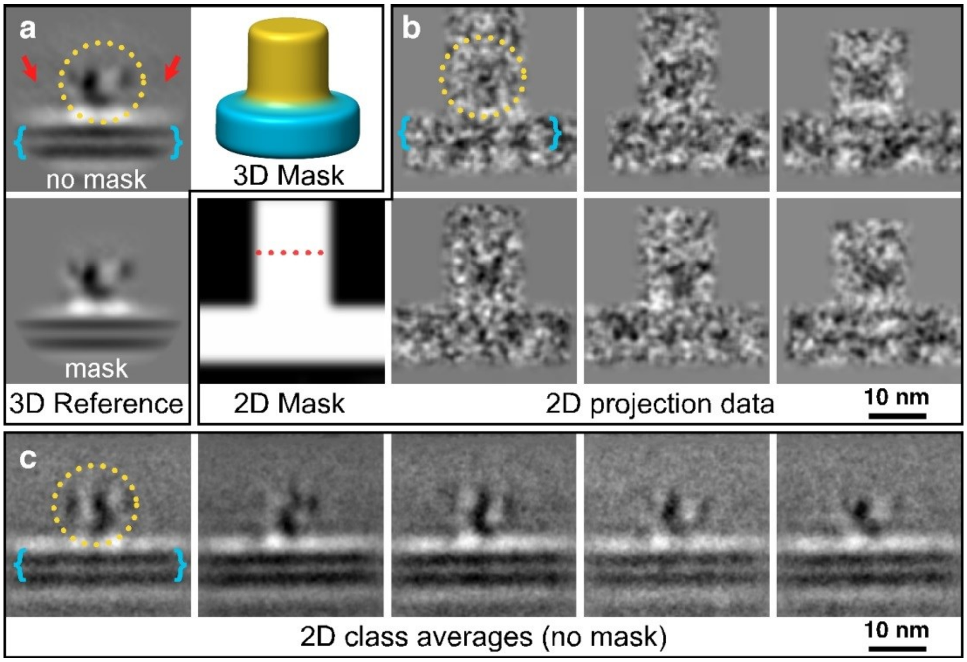

Supplemental Figure S2. 2D analysis of the Ebola virus GP spike. (**a**) Images of a 3D reference volume showing 2D projections without a mask, and with a mask, as well as a shaded surface representation of the 3D mask. The colours indicate the region that contains the spike (yellow), and the region that contains the viral envelope (blue). (**b**) Image of the "inverted T" shaped mask, and representative masked images, that contain the GP spike and virus envelope. (**c**) Final class averages generated by the projection match procedure. The GP spike is identified by a yellow circle, and the virus envelope is denoted by blue curly-bracket symbols.

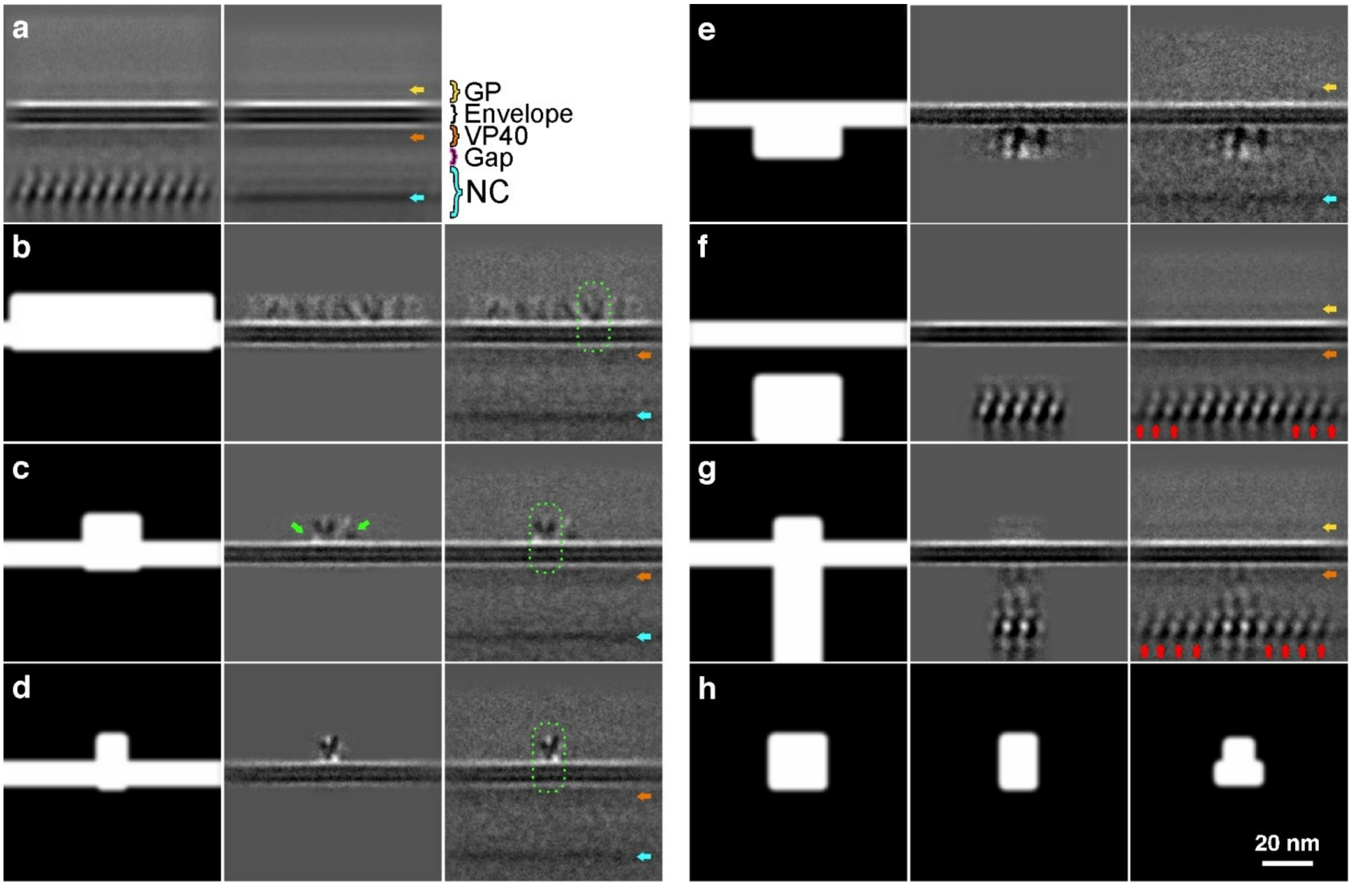

Supplemental Figure S3. Two dimensional image processing of half-diameter images of EBOV. (**a**) Image average of unmasked images (left), the right hand side image is a horizontally version of the image on the left. This process blurs the alignment of the nucleocpasid, with has an ordered periodicity, to avoid any bias in the subsequent analysis of the GP spikes, which have a variable spacing. These images show the relative location of the glycoprotein spikes (GP), viral envelope, VP40, and nucleocapsid (NC). (**b-g**) Processing of masked images including information specific to the viral envelope, and adjacent regions of the virion. Each three-image set is comprised of a mask (left), an average of the masked images (centre), and the total unmasked image averages (right). Panels **b-d** show the results obtained using masks that reduce the area of GP spike specific data analysed. The green arrows in (**c**) point to signal attributed to adjacent GP spikes, the blue arrows point to the density attributed to the NC, and the dotted green lines show the location of GP spike averages with “Y” shaped spike morphology. (**e**) Processing of masked images with information specific to the viral envelope and VP40. (**f**) Processing of masked images including only data regions specific to the viral envelope and the NC. The vertical red arrows in the right panel show the NC repeating pattern continuing outside of the masked region. (**g**) Processing of masked images including information specific to the viral envelope, VP40 matrix layer, and the NC. The vertical red arrows in the right panel show additional NC repeating pattern outside of the masked region. (**h**) Additional control masks, to test the effect of excluding the viral envelope from the 2D analysis, on the apparent spike structure: none of these masks produced good in-plane alignments. The coloured horizontal arrows on the right side of the all the images indicate the blurred densities corresponding to the GP (yellow), VP40 (orange), and the NC (blue) layers.

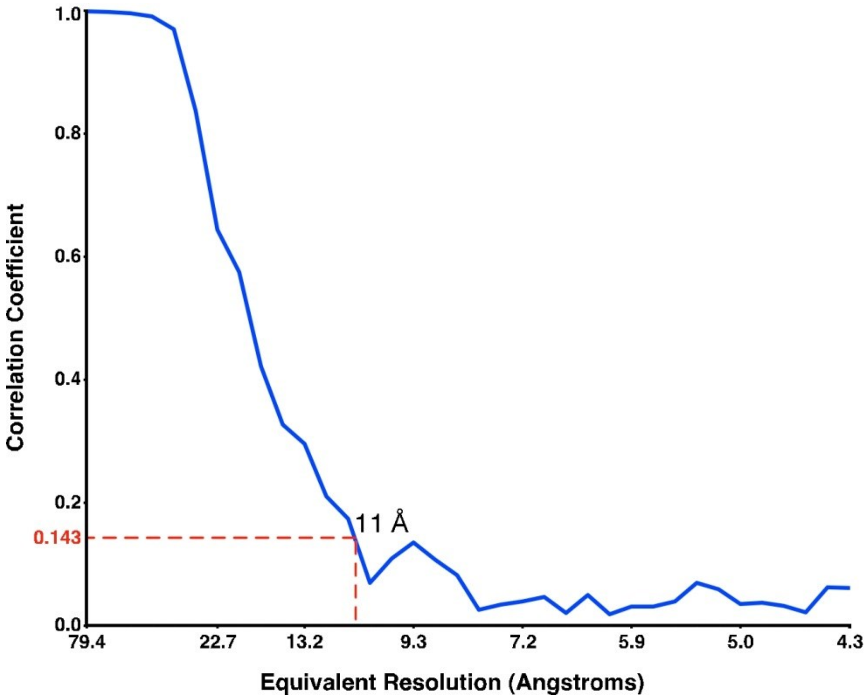

Supplemental Figure S4. Resolution of the EBOV GP spike structure. The Fourier shell correlation shows a value of 11A resolution at a coefficient of 0.143
